# 2555 Predictive cytological topography (PiCT): A radiopathomics approach to mapping prostate cancer

**DOI:** 10.1017/cts.2018.108

**Published:** 2018-11-21

**Authors:** Sean D. McGarry, Sarah L. Hurrell, Kenneth Ickzkowski, Anjishnu Banerjee, Kenneth Jacobsohn, William Hall, Mark Hohenwalter, Peter LaViolette, Amy Kaczmarowski, Tucker Keuter, Marja Nevalainen, William See

**Affiliations:** 1 Medical College of Wisconsin

## Abstract

OBJECTIVES/SPECIFIC AIMS: The objective of this study is to use machine Learning techniques to generate maps of epithelium and lumen density in MRI space. METHODS/STUDY POPULATION: Methods: We prospectively recruited 39 patients undergoing prostatectomy for this institutional review board (IRB) approved study. Patients underwent MP-MRI before prostatectomy on a 3T field strength MRI scanner (General Electric, Waukesha, WI, USA) using an endorectal coil. MP-MRI included field-of-view optimized and constrained undistorted single shot (FOCUS) diffusion weighted imaging with 10 *b*-values (*b*=0, 10, 25, 50, 80, 100, 200, 500, 1000, and 2000), dynamic contrast enhanced imaging, and T2-weighted imaging. T2 weighted images were intensity normalized and apparent diffusion coefficient maps were calculated. The dynamic contrast enhanced data was used to calculate the percent change in signal intensity before and after contrast injection. All images were aligned to the T2 weighted image. Robotic prostatectomy was performed 2 weeks after image acquisition. Prostate samples were sliced using a 3D printed slicing jig matching the slice profile of the T2 weighted image. Whole mount samples at 10 μm thickness were taken, hematoxylin and eosin stained, digitized, and annotated by a board certified pathologist. A total of 210 slides were included in this study. Lumen and epithelium were automatically segmented using a custom algorithm written in MATLAB. The algorithm was validated by comparing manual to automatic segmentation on 18 samples. Slides were aligned with the T2 weighted image using a nonlinear control point warping technique. Lumen and epithelium density and the expert annotation were subsequently transformed into MRI space. Co-registration was validated by applying a known warp to tumor masks noted by the pathologist and control point warping the whole mount slide to match the transform. Overlap was measured using a DICE coefficient. A learning curve was generated to determine the optimal number of patients to train the algorithm on. A PLS algorithm was trained on 150 random permutations of patients incrementing from 1 to 29 patients. Slides were stratified such that all slides from a single patient were in the same cohort. Three cohorts were generated, with tumor burden balanced across all cohort. A PLS algorithm was trained on 2 independent training sets (cohorts 1 and 2) and applied to cohort 3. The input vector consisted of MRI values and the target variable was lumen and epithelium density. The algorithm was trained lesion-wise. Trained PiCT models were applied to the test cohort voxel-wise to generate 2 new image contrasts. Mean lesion values were compared between high grade, low grade, and healthy tissue using an ANOVA. An ROC analysis was performed lesion-wise on the test set. RESULTS/ANTICIPATED RESULTS: Results: The segmentation accuracy validation revealed *R*=0.99 and *R*=0.72 (*p*<0.001) for lumen and epithelium, respectively. The co-registration accuracy revealed a 94.5% overlap. The learning curve stabilized at 10 patients with a root mean square error of 0.14, thus the size of the 2 independent training cohorts was set to 10, leaving 19 for the test cohort. DISCUSSION/SIGNIFICANCE OF IMPACT: We present a technique for combining radiology and pathology with machine learning for generating predictive cytological topography (PiCT) maps of cellularity and lumen density prostate. The voxel-wise approach to mapping cellular features generates 2 new interpretable image contrasts, which can potentially increase confidence in diagnosis or guide biopsy and radiation treatment.

